# Identifying women’s needs to adjust to postpartum changes: a qualitative study in Iran

**DOI:** 10.1186/s12884-022-04459-8

**Published:** 2022-02-11

**Authors:** Mahboobeh Asadi, Mahnaz Noroozi, Mousa Alavi

**Affiliations:** 1 Faculty of Nursing and Midwifery, Department of Midwifery, Isfahan (Khorasgan) Branch, Islamic Azad University, Isfahan, Iran; 2 Department of Midwifery and Reproductive Health, School of Nursing and Midwifery, Isfahan University of Medical Sciences, Isfahan, Iran; 3 Department of Psychiatric Nursing, School of Nursing and Midwifery, Isfahan University of Medical Sciences, Isfahan, Iran

**Keywords:** Needs, Adjustment, Postpartum, Qualitative study, Iran

## Abstract

**Background:**

The transition to motherhood is associated with stress because of extensive and rapid changes to which women need to be able to adjust. To help women adjust to postpartum changes, their needs during this period must be identified. Therefore, the present qualitative study explored the needs of women for adjusting to postpartum changes.

**Methods:**

In this study, 29 participants were selected through purposive sampling with maximum variation in Isfahan, Iran. Data was collected through in-depth individual interviews, daily notes, and field notes, and analyzed using conventional qualitative content analysis.

**Results:**

Data analysis led to the emergence of 5 main categories: “the need to receive social support,” “the need to receive a sense of worth from the husband,” “the need to compensate and improve the situation,” “the need to create socio-cultural changes,” and “the need for training.”

**Conclusions:**

According to the results of the study, in order to adjust to the changes in the postpartum period, and in addition to their efforts to compensate and improve the situation, women need to be supported by their husbands, family members and acquaintances, healthcare team, and society in various dimensions. Moreover, they should receive the information they need to turn the challenges of this period into an opportunity for growth.

## Background

Although motherhood is a pleasant and unique experience, the transition to motherhood is often accompanied by stress and tension because of extensive and rapid intrapersonal and interpersonal changes [1, 2] which occur in various physical, psychological, social, economic, and family dimensions. The woman who experiences these changes must be able to adapt [3, 4]. According to Lazvas and Folkman, adjustment is defined by evaluating personal resources and reaching an estimate as to whether the resources are sufficient or not and whether or not the situation is truly stressful, followed by entering a stage of confrontation and action. In other words, adjustment is synonymous with situation management [5]. After childbirth, women who successfully adapt assess their situation correctly and try to control it using the resources available to them. Those who do not find the appropriate resources suffer from stress and other difficulties [3]. Some widely recognized perinatal psychological health disorders, such as postnatal depression and postpartum psychosis, highlight the uniqueness of perinatal events and their potential impact on psychological health [6]. Studies have reported that postpartum mood disorders occur in 30 to 70% of women [1]. Depression is reportedly experienced in 13% of women. In such cases, other family members are also affected. A mother’s depression and psychological imbalance in the first weeks and months after childbirth can weaken the mother-child relationship and even lead to behavioral issues in the child [7, 8]. Davies et al. identified the need to address maternal depressive symptoms as distinct issues considering their differential effects on parenting behavior [9]. Other studies have also shown that the incidence of postpartum depression in women can cause depression and anxiety in their husbands [10, 11]. Postpartum psychological problems also have heavy economic costs for healthcare systems. A study in the UK estimated that depression, anxiety, and psychosis related to pregnancy and childbirth imposed costs of about 8.1 billion pounds annually on society [12]. However, with the appropriate adjustment, a woman can experience the time after childbirth as one of the best periods of her life and the life of her family, and the challenges that arise can be opportunities for a woman’s personal growth, increasing her knowledge, and better understanding herself and the formation of her identity [13, 14].

Women’s experiences in the postpartum period are influenced by social and cultural factors. Therefore, the studies in other countries may not be applicable to an Iranian population. Despite the large number of extensive surveys on maternity experiences in different countries, there are still numerous unknown aspects to this stressful stage. A review of research showed that only a limited number of studies have been conducted on women’s adjustment to postpartum changes in Iran, and the information is limited [15–17]. Appropriate policies and planning to improve women’s adjustment to postpartum changes and, consequently, increase their health cannot be established except through recognizing women’s needs in this period. As qualitative research is an approach to discovering and describing people’s experiences and giving them meaning and is used when there is a need to explain concepts and the relationship between them [18], the present qualitative study was conducted to explore the needs of women to adjust to postpartum changes.

## Methods

### Study design

This research with a content analysis approach [19] was part of an extensive mixed methods study that was conducted from August 2019 to January 2020.

### Settings, sample and recruitment

Participants included 17 women who gave birth and 12 healthcare providers (3 midwives, 3 gynecologists, 3 psychiatrists, and 3 psychologists) in Isfahan, Iran, who were selected by purposive sampling. Then, the selection of women who gave birth was continued using a strategy with maximum variation in terms of age, education, occupation, number of births, mode of birth, and time elapsed since giving birth. The selection of healthcare providers continued through a strategy with maximum variation in terms of length of work experience. Inclusion criteria were a willingness to participate in the study after giving informed consent, ability to express experiences, Iranian citizenship, the lapsing of 2 months to a maximum of 2 years from the birth of a live and healthy neonate, and the absence of major psychological disorders and chronic diseases. The inclusion criterion for healthcare providers, was having at least five years’ experience working as a healthcare provider. Participants were accessed through health centers, hospitals, and private offices of gynecologists, psychiatrists, and midwives. Participants were recruited by inviting them to participate via a face-to-face meeting or using phone calls [18]. Once enrolled in the study, none of the participants withdrew. The first author (M.A) had no previous relationship with any of the participants or centers. The demographic characteristics of the study participants are given in Tables 1 and 2.

**Table 1:** Demographic characteristics of participating postpartum women

Participant	Mode of birth	Time elapsed since giving birth (months)	Number of births	Educational level	Job	Age (years)
1	Cesarean	5	1	PhD	Employed	30
2	vaginal	15	1	High school degree	Housewife	28
3	vaginal	11	3	BS	Employed	41
4	vaginal	17	1	BS	Housewife	22
5	Cesarean	8	1	High school	Housewife	27
6	Cesarean	12	2	MS	Employed	35
7	vaginal	9	1	BS	Employed	33
8	Cesarean	6	1	High school	Housewife	32
9	vaginal	4	2	High school degree	Housewife	30
10	vaginal	2	2	High school degree	Housewife	19
11	vaginal	21	1	BS	Housewife	27
12	vaginal	18	1	High school degree	Housewife	20
13	Cesarean	10	1	BS	Employed	34
14	vaginal	7	2	BS	Housewife	36
15	Cesarean	6	2	PhD	Employed	37
16	vaginal	9	2	BS	Housewife	35
17	vaginal	3	3	High school degree	Housewife	36

**Table 2:** Demographic characteristics of other participants

Participant	Work experience (years)	Job	Age (years)
18	15	Midwife	43
19	24	Midwife	50
20	7	Midwife	35
21	35	Gynecologist	65
22	20	Gynecologist	50
23	20	Gynecologist	48
24	25	Psychiatrist	55
25	24	Psychiatrist	57
26	5	Psychiatrist	37
27	35	Psychologist	60
28	28	Psychologist	58
29	24	Psychologist	48

### Data collection

Semi-structured in-depth individual interviews [19], field notes, and daily notes were used to collect data. The first author (M.A), who had 7 years midwifery experience and was a Ph.D. candidate in reproductive health at Isfahan University of Medical Sciences, conducted the interviews. The other authors had previous interviewing experience and qualitative paper/report writing. Prior to data collection, the first author wrote down the initial preconceptions about the study topic based on her previous work experience and a literature review. Questions, prompts, and guides were provided, and this was piloted in one pilot interview. Interviews with postpartum women began with general questions like: “*Please explain how you felt after childbirth and the birth of your neonate?” “Since then, what needs have you felt regarding this?”* Then, the participants’ open and interpretive responses guided the process. Interviews with healthcare providers began with the general question, “*What do you think women who have given birth need to adjust to changes in the postpartum period? Please explain.”* Then, their open and interpretive answers guided the interview process. The time and place of the interviews were determined based on the desire of the participants. No one but the participant and the researcher was present at the interview. Each interview lasted between 30 and 90 min (45 min on average) and was recorded with a digital tape recorder. Interviews continued until data saturation was achieved. The first author (M.A) also recorded her observations of participants’ nonverbal behaviors during the interviews as field notes. She also asked women who gave birth to record and submit to her daily issues related to their needs in the postpartum period (daily notes).

### Data analysis

Data analysis was performed manually using the conventional qualitative content analysis method [20]; no software was used. The interviews were transcribed verbatim by the first author (M.A) and then read repeatedly to gain a full understanding of them. Then the sentences and phrases were coded. Coding was done by the first author (M.A) with a subset of 10% of the transcripts coded independently by the second author (M.N) using the developed coding frame. After the codes were formed inductively, similar codes were merged, and those with similar meanings were grouped together to form “sub-categories.” By comparing the sub-categories with each other, the categories that were conceptually related to each other were placed in a main category.

### Rigor and trustworthiness

To ensure the rigor and trustworthiness of data, four criteria are suggested: credibility, confirmability, dependability, and transferability [21]. Various methods were used to validate the data, including in-depth interviews at different times and places and a combination of several data collection methods such as in-depth interviews, field notes, and daily notes. Participants were selected with maximum variation strategy (in terms of age, education, occupation, number of deliveries, type of deliveries, and time elapsed since delivery). In other sessions, the transcripts and coded interviews were shared with three participating women who gave birth, and their final opinions were summarized so that the review could be done by the participants (member checking). The opinions of three experts were used to reconcile and ensure the consistency of the data with the statements of the participants. To increase transferability, the findings of the study were presented to three non-participating women with similar characteristics as the women who had given birth to judge the similarity of the study results with their experiences.

### Ethical approval

Approval of the research was obtained from the Ethics Committee of the Vice Chancellor for Research of Isfahan University of Medical Sciences (approval code: IR.MUI.RESEARCH.REC.1397.476). In the present study, informed consent, anonymity, confidentiality of information, and the right to withdraw at any time were observed. The reasons for the study were explained prior to each individual interview.

## Results

Data analysis resulted in 56 codes, 11 sub-categories, and 5 main categories. The main categories comprised “the need to receive social support,” “the need to receive a sense of worth from the husband,” “the need to compensate and improve the situation,” “the need to create socio-cultural changes,” and “the need for training” (Table 3).

**Table 3:** Results of the data analysis

Main category	Sub-category	Code
The need to receive social support	Receiving emotional support	Disappearance of the feeling of being alone in the postpartum period
		Being in the crowd and reducing worries
		A sense of encouragement followed by a sense of understanding the situation
	Receiving help and care	Receiving help in performing daily tasks
		Receiving care for faster recovery after childbirth
		Receiving care to build physical ability to breastfeed and perform maternal duties
The need to receive a sense of worth from the husband	Receiving love and affection	Receiving love from husband
		Receiving verbal appreciation from husband
	Receiving emotional approval from the husband	Confirmation of being beautiful by husband
		Confirmation of being good mother by husband
The need to compensate and improve conditions	Efforts to improve physical condition	Doing exercise
		Searching for information on beauty and diet
		Adherence to a healthy diet
	Efforts to improve social status	Modeling successful mothers in pursuing goals and aspirations
		Plan for a better social future, after relieving postpartum responsibilities
The need to create socio-cultural changes	The change in social norms and gender relations	The need for husband’s participation in child care
		The need for husband’s participation in housekeeping
	Adoption and implementation of supportive laws	The need to improve maternity and breastfeeding leave
		The need to enforce paternity leave
The need for training	Receiving information about the child’s health	Reducing anxiety after knowing about the child’s health
		Increasing self-confidence after awareness of the child’s health
	Receiving information about the mother’s health	Reducing anxiety after knowing how to return to physical changes after childbirth
		Reducing anxiety after raising awareness about health symptoms and danger signs
	Receiving information about how couples communicate	Increasing the cooperation of husband by being aware of the duties and responsibilities of the father
		Increasing understanding and empathy of the husband by being aware of psychological changes in the postpartum period
		Creating mutual understanding in couples after recounting the needs of both (husband and wife)

### The need to receive social support

Participants believed that women in the postpartum period need the support of others to adapt to new circumstances, and this support should cover a variety of factors, such as emotional support, care, and cooperation in doing tasks. This main category consisted of two sub-categories.

#### Receiving emotional support

Women who had given birth believed that they needed the support of others (such as family members, friends, and healthcare providers) after giving birth to reduce anxiety, relieve their loneliness, and create comfort and peace. They found the existence of such “supporters” very helpful and reassuring. One postpartum woman said:

*"… After giving birth, a woman has a lot of worries and looks for support or people who somehow assure her." (P15)*

#### Receiving help and care

Most of the participants stated that women after childbirth need the help and care of others (such as family members, friends, and acquaintances) to recover and return to their prior physical strength and perform their duties. Some of the women who had given birth mentioned the existence of a “helper” as their most important need during this period. One postpartum woman said:

*"… Lack of help is my biggest problem in this period. I am alone to do all my duties, and I get very tired! It would be great if there was someone to help me."(P13)*

Participating healthcare providers believed that not receiving help and care in the postpartum period would cause the mother to feel lonely and, as a result of the stress, disrupt the mother’s adjustment to postpartum changes. One of the psychiatrists stated:

*"… Taking care of a child in the first two years of life is a 24-hour duty, and a mother should have some people to help her during this time. In the absence of this source of help, a mother feels alone in the face of these responsibilities, and this feeling can be very stressful and disrupt the mother’s adjustment.” (P24)*

### The need to receive a sense of worth from the husband

Participants’ statements indicated that women in the postpartum period need their husbands to express more love and affection. Receiving the approval of the husband can reduce their worries in many cases. This main category consisted of two sub-categories.

#### Receiving love and affection

The narrations of the participating women indicated that the behavior of their husbands during this period was very important to them. They needed to be appreciated and loved by their husbands for giving children to them (they considered the baby as a divine gift) and for enduring the hardships of pregnancy and postpartum. One postpartum woman said:

*"... When my husband loves me and thanks me for doing my motherly duties, my tiredness decreases, I feel relaxed and better. I feel like there is someone for whom I have value, and he appreciates my efforts." (P6)*

The need for women who have given birth to receive love and affection from their husbands is such that if this expectation is not fulfilled, they express grief and dissatisfaction. One postpartum woman said:

*"I really expected my husband to somehow appreciate and thank me after giving birth. Didn't I give him a baby? But my husband did nothing, not even the smallest gift or verbal thanks." (P5)*

One gynecologist stated:

*“… Women in the postpartum period need more expressions of love from their husbands. By doing so, they think they are valuable to their spouse.” (P21)*

#### Receiving emotional approval from the husband

The statements of the participating women indicated that it was very important for them to receive approval from their husbands in the postpartum period. They narrated that with the approval of the husband, their worries about the changes in their body were eliminated and their self-confidence was increased. One postpartum woman said:

*"… Although I got fatter, I do not feel ugly at all, because my husband keeps telling me that I have become more beautiful. I have good self-confidence and, thank God, I do not worry about my appearance." (P3)*

Participating women also narrated that after receiving confirmation from their husband about their good and proper performance as a wife and as a mother, their worries were replaced by calm and confidence. One postpartum woman said:

*"My husband's approval is very important to me! When I ask him if he thinks I am a good mother or a good wife and he answers that I am the best wife and mother in the world, I am very happy, and I really feel like I am the best wife and mother in the world, and I calm down." (P11)*

### The need to compensate and improve the situation

The participating women stated that many changes in the postpartum period have taken them away from their ideals. Therefore, many of them sought to compensate with better adjustment to the new conditions. This main category consisted of two sub-categories.

#### Efforts to improve physical condition

Statements by some of participating women indicated that physical changes in the postpartum period such as overweight, loose abdominal skin, the presence of “stretch marks” on the abdomen and thighs, and sagging breasts are not pleasant for them. To return to pre-pregnancy and pre-partum fitness, they turned to activities such as exercise, dieting, and obtaining information, so as to get closer to their ideal body and solve their worries in this regard. One postpartum woman said:

*"… Losing the beauty of my body has become my psychological concern, and I am researching in the field of exercise, diet, and ways to eliminate “stretch marks” on my skin. Of course, I have been exercising for some time, and I want to continue exercising in the same way to achieve the desired result." (P4)*

One of the midwives said:

*“Major concerns of women in the postpartum period are weight loss and fitness. For this reason, they are constantly looking for effective solutions to achieve the desired result.” (P18)*

#### Efforts to improve social status

The narrations of the participating women indicated that leaving their job due to childcare and neglecting individual aspirations are other worries and psychological preoccupations. They used to plan for the future and follow the example of other successful mothers to compensate for the conditions that occurred in the postpartum period. One postpartum woman said:

*"… I went to music and language classes before giving birth, but now I do not have time, and this upsets me, and I am worried that I will fall behind in my dreams. But now that my niece has grown up, my sister is going to music class. I also decided that after my child is 2 years old, I will definitely continue my classes." (P2)*

### The need to create socio-cultural changes

Participants believed that due to the existence of gender roles in the family and society, the placement of duties and responsibilities in the form of “male” and “female” roles prevents men from supporting their wives in the postpartum period. Moreover, family protection laws for the postpartum period are not sufficient or satisfactory, and changes in this field were considered necessary. This main category consisted of two sub-categories.

#### The change in social norms and gender relations

The narrations of some participants indicated that in many families, maternal responsibilities and housework are among the duties and roles of women, and men do not consider these tasks to be their duty or responsibility. This issue deprives women of their husbands’ participation and cooperation in many matters in the postpartum period. Many participants considered the patriarchal culture of the families to be wrong and very annoying, and they wanted to change it. One postpartum woman said:

*"… When I ask my husband for help, I have to put a lot of energy into convincing him to do the work and finally thank him for his help. He considers almost all household chores and childcare to be the inalienable duties of a woman, and this belief is very annoying." (P7)*

#### Adoption and implementation of supportive laws

The narrations of the participating women showed that in cases where the husband’s working conditions do not allow him to cooperate and support the woman in the postpartum period, there is a need to provide more support for postpartum women through the implementation of laws such as “paternity leave”. One postpartum woman said:

*"… I am employed, and I thought that my husband could take 2 weeks off and help me, but they did not give my husband leave after childbirth! In my opinion, pregnancy is very difficult for employees unless there is enough supportive law." (P1)*

Working postpartum women also expressed dissatisfaction with the way the existing laws (maternity leave, hourly breastfeeding leave) were enforced. They narrated the need to reform or enforce the law properly as much as possible to support women who give birth. One postpartum woman said:

*"… After giving birth, I realized that my salary is paid with a deduction of one third and at intervals of 2 months! During the difficult economic situation when I was repaying the loan, I had to return to my job when my child was three months old and leave my child with my mother, and I am upset about this situation." (P7)*

One of the midwives also said:

*"... Maternity and breastfeeding leave are not enough for working mothers, and I think these rules should be amended so that the mother feels more relaxed and less worried during this period." (P19)*

### The need for training

Participants believed that women in the postpartum period have various educational needs regarding their health and their child’s health as well as how couples communicate. They narrated that meeting these needs can play an important role in women’s adjustment to postpartum changes. This main category consisted of three sub-categories.

#### Receiving information about the mother’s health

Participants’ statements indicated that women had many questions in the postpartum period about such topics as physical changes in the body and how it returns, health risk signs, how to take medicine, proper nutrition, methods of contraception, and sexual function. They believed that meeting these educational needs could greatly reduce women’s stress and anxiety and boost their self-confidence. One postpartum woman said:

*"... I'm very worried about my physical condition, whether it will return to what it was before pregnancy or not, and I would like to know what I should do to make these changes come back faster. I would like to know about breast problems during this period, the medicines I take, or anything else." (P8)*

One of the midwives also said:

*“... In the postpartum period, women have questions about danger signs, diet, family planning, sexuality, and other things.” (P20).*

#### Receiving information about the child’s health

Participants’ statements indicated that child health is one of the most important concerns of women in the postpartum period. However, by receiving education and increasing their awareness on how to care for their children and maintain and promote their health, their self-confidence increases and their worries decrease. One postpartum woman said:

*"… My child's health is my most important concern! When something happens to my child and I am not sure whether the situation is normal or not, I do not have peace of mind. I do research on the Internet, I ask my mother, I go to the doctor's office, and my anxiety will not diminish until I know the reason and make sure it does not endanger my child. "(P12)*

One of the gynecologists stated:

*“… Adequate education and information during the postpartum period are very important; they increase the mother's self-confidence and greatly reduce her worries about the child’s health.” (P23)*

#### Receiving information about how couples communicate

Some participants narrated that as women’s responsibilities increase with the birth of a child, many of the couples’ interactions in the postpartum period are affected by the new circumstances. Because women spend a lot of time caring for their children, they cannot spend as much time as before communicating and interacting with their husbands. On the other hand, women also need their husbands to help them with empathy, cooperation, and participation in doing things. Therefore, such things can cause problems between couples. One postpartum woman said:

*"… With the birth of my child, I do not have time for my husband as before. On the other hand, I expect him to spend more time cooperating, understanding, and empathizing with me. He is also dissatisfied with me and says that with the birth of the baby, I do not love him as before." (P16)*

Participants believed that couples should be educated on how to communicate properly with each other in the postpartum period and maintain their communication balance through mutual understanding of each other’s new needs. One of the psychiatrists stated:

*"... Well, naturally, many couples do not have enough awareness of the needs and proper communication behavior in this period, and it is the duty of the health team to help them with the right training." (P25)*

A postpartum woman said:

*"... I think we both need advice and training, because neither of us knows the right relationship in this period." (P16)*

## Discussion

The aim of this study was to explore the needs of women in adjusting to postpartum changes. The results showed that women who understand the shortcomings in the process of adjusting to postpartum changes need the following to eliminate them: to receive social support, to receive a sense of worth from the husband, to compensate and improve the situation, to create socio-cultural changes, and training.

Participants in the present study believed that women in the postpartum period need social support to adapt to new conditions. It is believed that after childbirth, because of physical problems (such as fatigue, abdominal pain, breast problems, etc.), women not only have various responsibilities (babysitting, marital duties, caring for other children, social duties, etc.), they also become physically and psychologically vulnerable [7]. Failure to bestow “perfect” motherhood can provoke a range of disordered constructions of love, and important consequences of the “good mother” discourse include increased maternal anxiety. This can manifest as maternal ambivalence and mother-infant attachment issues which, in turn, may have profound, lifelong implications for the psychological health of both mother and child [22]. Therefore, mothers need supporters who will take care of them in these situations, help them in doing their duties, and be able to support them psychologically in critical moments [5]. Ospina et al. defined the postpartum period as the period in which mothers need comprehensive support [13]. Dennis et al. and Kassam both mentioned the lack of adequate support from others as the main cause of perceived postpartum stress [23, 24]. In a study by Yu et al. a lack of social support was significantly associated with postpartum depression [25].

In the present study, the participating women not only needed to receive support from others, they also had certain expectations from their husbands in terms of receiving a sense of worth. It has been shown that with the birth of a child, the mother sees her place in the universe differently and considers the birth of a child to be the greatest gift from God [7]. In the present study, women who had given birth felt that it is their right to be appreciated by their husbands and to receive love and affection, because they gave a divine gift to their husbands and endured hardships and problems related to the care of the child. They also reported that they have worries about changes in their body appearance and their functions and duties, which are reduced with the emotional approval of their husbands. In the study of Alves et al. the supportive role of men in reducing depression in women with low self-esteem was emphasized [26]. Dennis et al. mentioned the emotional support of the husband as a factor in increasing the ability of women to adjust to stressful events after childbirth [23].

In the present study, the participating women needed to plan and take action to return their bodies to their pre-pregnancy forms as well as to pursue their goals and aspirations. Exercise, following a healthy diet and following the example of successful mothers were among the programs and actions they needed. In the study of Ospina et al. exercise and following a healthy diet were mentioned as contributing factors to restoring the physical condition and increasing the morale and confidence of women [13]. Ashaba et al. showed that observing other mothers who have overcome their problems in the postpartum period can help women overcome and adjust to postpartum challenges [5].

In the present study, participants in some cases considered socio-cultural changes as a prerequisite for their adjustment to postpartum changes. They were dissatisfied with gender stereotypes in Iranian culture that considered housework and childcare “feminine”. It was found that in families where the husband is heavily involved in gender roles and is unwilling to help or assist the woman who has just given birth in performing her duties, the wife loses one of her most important sources of support [1]. Figueiredo and Conde also showed that in different cultures, women’s adjustment problems to postpartum changes are varied, which is due to the different share of parental responsibilities [27]. Therefore, changes in social norms and gender relations are necessary for women to have the support, cooperation and participation of their husbands in the postpartum period. In addition, participants in the present study considered the adoption and implementation of supportive laws on childbirth and breastfeeding necessary. Ospina et al. also considered the adoption of supportive laws on childbirth and breastfeeding to be a prerequisite for adjusting to new maternal conditions [12]. Zare et al. also showed in their study that receiving support from the husband is possible, if the husband can be physically present in the house and the existence of “parental leave” for the husband be a national law. Also, in cases where a woman is concerned about her social and professional roles, the existence of reliable centers such as preschools can help her [28]. Therefore, because the needs of women in the postpartum period are very diverse, laws should be enacted in order to protect them as much as possible.

Participants in the present study mentioned the need for education on the health of postpartum women and their children as well as on how couples communicate. Given that postpartum changes are so widespread, unawareness in any case can pose a risk or concern for mothers, infants, and their family relationships [29]. In a study by Entsieh and Hallström, awareness was mentioned as one of the basic needs for adjusting to changes after childbirth and transition to motherhood. According to these researchers, positive psychological readiness can reduce depression and anger by changing the level of self-efficacy and feeling of better control. Sections required in postpartum training can include parental confidence, parenting and communication skills, how to receive specialized support and health facilities, possible changes in marital relationships and how to deal with them [30]. In Iran, midwives and obstetricians play important roles in the counseling and health education for women. They are responsible for maintaining and promoting a mother’s health by providing high quality care and accurate information. Golyan Tehrani et al. showed that postpartum care education based on the educational needs of mothers compared to routine education increased puerperal care knowledge [31]. Therefore, meeting the educational needs of women can play an important role in the process of their adjustment to changes after childbirth [32].

### Strengths, limitations, and future directions

The findings of the present study can help in designing the necessary interventions to improve postpartum women’s health by presenting an image regarding women’s needs to adjust to postpartum changes for the first time. Limiting the choice of postpartum women who have passed 2 months to a maximum of 2 years from the birth of a live and healthy neonate may be one of the limitations of the present study. The results of this study could be used by healthcare providers in the field of reproductive health for related consultations, education, and care for postpartum women. Moreover, they could improve healthcare services provided to mothers and their children. The results of this study are also expected to be used as a basis for future research in the field of women’s adjustment to postpartum changes and will lead to the identification of newer research areas.

## Conclusion

The results of the present study showed that to adjust to the changes in the postpartum period, women, in addition to their efforts to compensate and improve the situation, need to be supported by their husbands, family members and acquaintances, their health team, and society in various dimensions. They should also receive the necessary information to reduce their worries and enjoy this period. Healthcare providers can play an effective role in health planning and service delivery by being fully aware of these needs, by promoting the health of mother and child, and by supporting family and society.

## Data Availability

The datasets generated and/or analysed during the current research are not publicly available as individual privacy could be compromised but are available from the corresponding author on reasonable request.

## Abbreviation

UK: United Kingdom
